# A qualitative study of phenomenology of perspectives of student nurses: experience of death in clinical practice

**DOI:** 10.1186/s12912-022-00846-w

**Published:** 2022-03-29

**Authors:** ShiShuang Zhou, LiZhen Wei, Wei Hua, XioaChong He, Jia Chen

**Affiliations:** 1 Department of Nursing Aministration, School of Nursing, Army Medical University, Chongqing, China; 2 XiangYa Nursing School of Central South University, 172 TongZiPou Rd, Yuelu District, Changsha, Hunan 410000 China; 3 JiangNing Hospital, Nanjing, China

**Keywords:** Death experience, Death education, Nursing students, Qualitative analysis

## Abstract

**Aim:**

To describe the experiences of student nurses in confronting the death of their patients, and to understand how they cope with these events and to what extent there are unmet needs that can be addressed in their trainings.

**Methods:**

Semi-structured interview method was used to collect data from Chinese nursing students and then Colaizzi’s seven-step analysis method was applied to identify recurrent themes in their responses to patient deaths. We listened the tape repeatedly combined with observations of their non-verbal behaviors, then transcribed them with emotional resonance, and entered them into Nvivo. After that, we extracted repeated and significant statements from the transcriptions, coded, then clustered codes into sub-themes and themes which were identified by the comparation with transcriptions and re-confirmation with our participants.

**Results:**

After confirmation from the interviewees, five themes emerged: emotional experience, challenge, growth, coping and support.

**Supplementary Information:**

The online version contains supplementary material available at 10.1186/s12912-022-00846-w.

## Introduction

Death is a sensitive topic in the majority of cultures [1, 2]. Particularly in Chinese traditional culture, people regard death as taboo and ominous, and they usually avoid talking about it. However, death is an inevitable and natural part of life.

Nurses take on a profound responsibility to look after dying patients in clinical settings. Moreover, with the increase in elderly populations in China, the need for medical care keeps growing. When providing this care, nurses have to face patients’ sudden or expected death. Researchers have identified that excessive exposure to the death of patients leads to compassion fatigue for nurses [3]. Additionally, when nurses actually experience patients’ deaths, some negative feelings including guilt, depression, frustration, sadness, incompetence, helplessness and distress come over them [4–7]. Hiding feelings and working continuously are common coping strategies they choose, as they are worried that showing their feelings may influence other’s perceptions of their professionalism [4, 7]. These negative emotions can decrease the quality of care delivered and increase attrition rates [7, 8]. Cevik and Kav have found that nurses’ negative attitudes toward death are associated with lack of education [9], and nurses also claim that they do not have adequate training, support or preparation regarding to death [10, 11]. Becoming qualified nurses, sufficient training related to death and supports to deal with death are required [12, 13] .

## Background

Nursing students are being prepared for future nursing careers. Some studies have shown that undergraduate students should be prepared for accompanying death (Munoz-Pino, 2014). Before they become qualified nurses, they need training in clinical practice settings where they may encounter patients’ deaths. However, most nursing students’ attitudes toward death include escape, death anxiety and fear as shown in previous quantitative studies [14–16]. Students’ anxiety may come from their roles of caring [17], handling the dead body and lacking preparation [18]. To our knowledge, most studies discuss nursing students’ feelings after patients’ deaths and in particular the first-time nurses encounter a death experience in a patient [12, 15, 16], The first experience may not be as memorable for some students as later experiences. Therefore, we decided to focus on the experience of death that created the strongest memories. These most memorable experiences will cause vivid memories and effects that persist. Our study will identify nursing students’ the most memorable death experience in clinical practice, with attention to the influence of this experience on their general outlook as nurses, the coping skills they employ to deal with such experiences, and their unmet needs. We hope to provide information that will assist educational faculties and clinical teachers about how they can assist nursing students who experience death in the clinic setting.

## Methods

### Aim

How nursing students remember and make sense of their most memorable experiences of patients’ deaths, in particular those which have had great and persistent effects on them.

### Design

Phenomenology both as an philosophy and research method is a valuable tool for researchers to understand the experience of people and “what” is hidden in those experiences [19]. We conducted our qualitative study using an interpretative phenomenological analysis approach. This approach, which derives from phenomenology and hermeneutics, combines psychological, interpretative and idiographic components. It has been widely used in many areas, for example pedagogy, medicine, and psychology [20, 21]. Participants’ experiences, understandings, perceptions and views are the main interests of this approach [22]. The interpretative phenomenological analysis method needs the collaboration both of researchers and participants to understand the experience, and it has been called a double hermeneutic as analysts attempt to make sense of their own experience and those of the research participants simultaneously. This study adhered to the checklist of COREQ (Supplementary File 1).

### Sample

Participants were recruited through purposive sampling from four primary hospitals of Hunan province in China between 20 March 2019 and 15 May 2019. We issued research recruitment notices via the Internet to the nursing students being trained in those hospitals. The inclusion criteria were: (1) Nursing students in clinical practice or had just finished their practice a month ago. (2) Nursing students who had clear memories of their experiences of patients’ death during their clinical practice. Participants who satisfied all inclusion criteria were given information about this study including its purpose, method, and the promise of confidentiality. They had right to choose whether to participate and drop out. The participants in this study had no prior relationships with the researchers, as friends or in their training. The final sample size was 19 nursing students (Table 1).

**Table 1:** Characteristics of participants

N	Education background	Age	Sex	Practice time (Month)	Area of residence	Religious faith	Received palliative courses
N1	A	18	Female	9	R	No	Yes
N2	B	22	Female	6	U	No	Yes
N3	B	20	Female	7	U	No	Yes
N4	A	19	Female	9	R	No	No
N5	B	21	Female	8	U	No	No
N6	A	19	Female	9	R	No	Yes
N7	A	18	Female	9	U	No	Yes
N8	A	20	Female	7	U	No	Yes
N9	B	21	Female	9	U	No	Yes
N10	B	21	Female	9	U	Yes	No
N11	B	22	Female	9	U	No	Yes
N12	B	18	Female	9	U	No	Yes
N13	B	18	Female	9	R	No	Yes
N14	B	20	Female	7	R	No	Yes
N15	B	21	Female	6	U	No	Yes
N16	B	21	Female	7	R	No	Yes
N17	B	23	Female	9	R	No	No
N18	B	20	Female	9	R	No	No
N19	B	21	Female	7	U	No	No

A:Junior College; B: Bachelor

R:Rural area; U:Urban area

### Data collection

A semi-structured interview method was used. An interview outline (Table 2) was based on literature review and experts’ opinions. Any additional themes not included in the initial outline, were added during the interview. Moreover, the participant’s answer to one question could be a guide to asking other questions. The main interviews were conducted with individual participants in quiet and undisturbed university psychological counseling rooms. After obtaining permission agreements from nursing students, all interviews were audio record and the researchers made field-notes to describe the sounds, tones, expressions and postures of the participants. Each interview lasted between 60 and 90 min. At the completion of 19 interviews the team established that no new information was being introduced and we ceased data collection.

**Table 2:** Interview guideline

**Questions**	
1. What comes to your mind when you hear the word death of a patient?	
2. Have you ever thought about patients’ death during your clinical practice? (If “yes”, ask their feelings and thoughts).	
3. Tell me about your experience with the patient’s death.	
4. Could you describe one of patients’ death scene that impressed you most?	
5. What were your feelings at patients’ death time?	
6. What were your feelings after a patients’ death?	
7. Did you have any changes after experiencing patients’ death? (If “yes”, ask their changes in thoughts and daily life)	
8. What did you do to cope the death experience?	
9. What did you need after experiencing the most impressive patients’ death?	
10. Do you think there is another question I should have asked and do you have a question for me?	

### Data analysis

Data analysis was based on Colaizzi’s seven-step analysis method (Table 3) which has been shown to be rigorous and robust [23]. Colaizzi’s seven-step analysis method provides researchers with clear, logical and sequential steps [24, 25]. Our analysis included non-verbal responses recorded in field-notes to help the investigators understand their verbal expression and the deeper meanings of their experiences. Above all, our analysis synthesized phenomenological description with our own empathic interpretations.

**Table 3:** Colaizzi’s seven-step procedure of data analysis

**Steps**	
**1.** Listen to the tape time and again and transcribe them into transcriptions, then read their words repeatedly combining with the non-verbal communications till feelings resonate.	
**2.** Extract Significant statements which are relevant to the death experience of nursing students from each transcript.	
**3.** Formulate meanings from significant statements and code them. Codes should be approved by all researchers.	
**4.** Cluster these codes.	
**5.** Confirm findings and identify difference by comparing transcriptions and sub-themes, themes more than once.	
**6.** Describe the theme and essence of nursing students’ experience by their own statements to insure the validity of study.	
**7.** Return each transcript and result to participants to affirm the findings.	

### Rigor

All researchers had learned about qualitative research methods and interview techniques and had interview experience. The interview outline was approved by three experts, including a professional psychologist, a nursing teacher who briefs student nurses about death experiences, and a clinical nursing teacher. In order to collect comprehensive data, another interviewer observed the interviewees’ expressions and asked supplementary questions to confirm the feelings evoked during the telling of their experiences with death and views appropriately.

Following to the seventh procedure of Colaizzi’s analysis method, we sent participants transcripts for their comments and suggestions. In data coding stage, two researchers coded independently to ensure consistency. If there were any differences, the third researcher would determine the final coding. We interviewed the participants and analysed the data using their mother language, Chinese. In reporting the results, nursing students’ words were translated into English and The Revised Standards for Quality Improvement Reporting Excellence (SQUIRE 2.0) (Supplementary File 1) were applied. We confirmed that all methods were carried out in accordance with relevant guidelines and regulations.

### Ethic

This qualitative study was found to be of minimal risk and was approved by Institutional Review Board (IRB) with IRB Approval Number: 2019020. As death is a distressing topic for the participants, the interview would be stopped if we found interviewees with uncontrolled and disturbing emotional responses. One of interviewers is a professional psychological consultant who could offer supports to the participants. We left contact information for a professional psychological team to support the interviewees who needed help after the interview.

## Results

Data saturation was reached after interviewing 19 interns whose information can be seen in Table 1. Their mean age was 20 years with a range from 18 to 24 and most of them (*n* = 13) had taken courses related to palliative care.

Eventually, five themes and thirteen sub-themes were emerged (Table 4) with the guide of Colaizzi’ analysis method. The emerged themes included: (1) emotional experience, (2) challenge, (3) growth, (4) coping, and (5) support.

**Table 4:** Themes and sub-themes of the most impressive death experience among nursing students

Themes	Sub-themes	Details
1. Emotional experience	Anticipations of experiencing patients’ deaths before entering clinical practice	Taboo Fear Unprepared Natural Acceptance
	Feelings witnessing a patient’s death	Scared Curious Startle Regretted Helpless
	Feelings after patient death	Dread Guilty Sadness Pity Upset Worry
2. Challenge	Cognition	Blind denial Obsessive thinking
	Somatization	Poor appetite Sleeplessness Visual illusion
	Habit	Irregular of diet Refuse to meat
	Profession	Empathy fatigue Emotion contagion Knowledge gap
3. Growth	Personal growth	Attention of health Awareness of cherish
	Professional growth	Life responsibility Professional identity
4. Coping	Exploratory method	Sharing with trust one Writing down
	Avoidant method	Do not remind death experience Transformation to other things Avoidance
5. Support	Clinical teachers	Give ear to students Provide experience relating to death Provide positive coping methods
	Education	Knowledge about death Skills to deal with death

### Emotional experience

The focus of this project was on nursing students’ the most memorable experience with the death of a patient and we found that the content of their feelings varied from the period in relation to time of death. These included anticipatory imaging of patients’ deaths, witnessing death and dealing with the aftermath of witnessing a patient’s death.

#### Anticipations of experiencing patients’ deaths before entering clinical practice

We asked how they felt when, prior to clinical practice, they imagined patients’ deaths. Some students had never thought of death before, and therefore had not prepared for a death experience.. Several students expressed taboo and fear, with no confidence about how to deal with death. Only one student thought death as an natural event. In the culture of China, people rarely use words related to death and dying in normal discourse. For many Chinese, discussing death is regarded as taboo. Nursing students in China will learn postmortem care in basis nursing courses, but that subject takes less than 45 min class time. It was understandable that most nursing students have these reactions when asked to imagine a patients’ death.

“In my family, we never talk about death, it is taboo”---N2

“I can’t understand Postmortem Care. Traditionally, death is not such a good thing .”----N13

“ I felt terrible when imaging that I would face death patients before entering clinical practice, ”---N19

“I think that I can't cope with it, I can escape it, I don't want to encounter it, and it will make people feel uncomfortable.”---N2

“I think it is normal for a person to be born, grow up and die; sickness and death, it’s a nature thing. So I will feel ok if I face death patients in clinical practice” ----N1

#### Feelings witnessing a patient’s death

A majority of students were frightened and helpless. Students voiced curiosity about how a dead body changes physically, but they were startled when they saw it. Nursing students’ feelings varied by age of a patients’ death and their relationship with the deceased. For younger the patients, they expressed strong feelings of pity. When they experienced the failure of an attempt to save a patient’s life, they felt helpless or expressed regret. As nursing students, they also had curiosity about the changes that occur after death and wanted to confirm the knowledge they got in class. Even students who had never cared for the patient felt scared or startles when just saw the body. When they had taken care of that patient, they experienced sadness and difficulty to say goodbye, as intimacy had developed between nursing students and patients.

He was just a baby that he had not yet enjoyed the beauty of the world, I want to take one last look at him.---N07

I felt nothing but...Umm...sort of pitiful because he was such a young man, only 27 years old! ”---N16

I saw a man lying there with a piece of cloth on his back even though I was just going to pick something up next to him, but I was really scared because that was really scary.”----N16

“I was just curious to know whether the state of death was similar to what we had learned. So I went to touch his hand. I was also very startled. His eyes were not closed...”---N11

“When I witnessed a patient struggling, I could do nothing but stand by and watch, which made me feel so helpless. ”---N12

“The patient didn't come back, he should have been able to come back, and I felt very guilty that I didn't do a good job”---N4 (A student has done cardiopulmonary resuscitation for the patient)

#### Feelings after patients’ death

The nursing students’ feeling of fear towards death was constant from the moment of a patient’s death to a period that depended on the circumstances of death. Even though a patient’s body had been moved to another location, the place of death could be dreaded long afterwards. After observing that poorer patients gave up their treatments due to high medical costs, some nursing students keenly felt the unfairness that some should die for lack of money while others with more resources could live. Patients’ deaths also trigger nursing students’ worry toward their family members.

“I was afraid to go to that ward alone every time I was on night duty. That thing really made me feel dread”(said with eyes changed more bigger) ---N11

“I just thought it unfair, someone can have chance to survive but he just can’t! His life cannot be decided by himself”---N8

“ And now, I sometimes worry that, what if my family or lover leaves? What shall I do? Where shall I go?---N15

### Challenge

The memorable death experience not only affects nursing students’ emotional experience but also other aspects of nurses’ lives, including cognition, somatizations, habits and profession. We identified these subjects as a theme called challenge.

#### Cognition

The patients’ death was obsessively in mind for some nursing students who would constantly recall the death. Thoughts questioning their capacity to handle future professional work were occurring in nursing students’ minds. Nursing students felt helpless when experiencing patients’ deaths and lacked the confidence to deal with such deaths in the future.

“After experiencing such a thing, I feel that I have no ability. I am afraid that I will work alone in the future and I don't know how to do it.”---N12

“I usually forgot my meals, lying in my bed, ignoring my roommates calling me for lunch, with my mind going blank except for the patient’s death”---N1

“...with the patient’s death always reflecting in my mind”---N17

#### Somatization

Mental experiences and states may become somatic symptoms. Students negative responses included poor appetite, sleeplessness and visual illusion. These somatization symptoms lasted about 1 week.

Some students said that they could see the dead patients’ soul in the enclosed space. Different theories of death may help to interpret their responses. Monism reckons that human beings are material, while the dualism believes that human beings are composed of soul and body, and that the soul can continue to survive after the death of human beings. Those who are in favor of dualism believe that the soul is released from the body after death and can roam more freely. Individuals’ opinion toward death was affected by traditional culture. For example, in China, we have Chinese Memorial Day to go to the departed loved ones’ grave to remember them. Perhaps the nursing students who had visual illusion for death patients believed the soul of dead patients still alive.

“... I lost my appetites.”---N11

“I was exhausted, but I couldn’t fall asleep.”---N17

“Sometimes, I can see the dead patients’ soul especially in enclosed space such as in an elevator”.---N4

#### Changes of habits

When a person has emotional and cognitive changes or even physical symptoms, his lifestyle may change, at least temporarily. Some nursing students said their living habits were changed by the experience of patients’ deaths. For example, their diet became irregular or they refused to eat meat. The deceased undergo physical decay after death when the temperature and the color of skin changes. So the dead body would look just like meat in supermarket and it is understandable that some nursing students had such reactions toward pork.

“These days I cannot eat and sleep as usual”---N11

“I couldn’t have meat for a long time. And I even felt scared when fresh meat came in sight.”---N6

#### Profession

Nursing students’ said that their emotion was strongly affected by the family members of the deceased. This can be called emotional contagion which is an psychological phenomenon where people “catch” feelings from one another like they would a cold. Because of “emotional contagion”, nursing students catch the emotions of the crying family members easily, which may impact their work performance and even daily life. However, a number of students indicated a fear of empathy fatigue over the long term after repeatedly experiencing patient deaths. Furthermore, as voiced by our interviewees, there are difficulties in transforming theory into practice, which is a challenge for nursing educators.

“I can’t control my emotions...I was deeply affected by them [death patients’ family members], then cried with them together .”---N10

“We will face patients’ death all the time as medical workers. We can’t save everyone. Firstly it makes me depressed, as for long term, when I experienced much, I am afraid that I will be indifferent and numb.”---N9

“Having experienced these losses, I found out that what I have learned from books cannot be applied correctly to the clinical circumstances, where you face things that are much more complicated than the example learned from textbooks.”---N7

### Growth

Growth refers to nursing students having positive responses and learning from the most memorable experience of encountering death. This theme had two sub-themes, personal and professional growth.

#### Personal growth

Several respondents reported changes in attitudes toward death. The passing of other individuals’ lives would make people think more about life. The death experience promoted nursing students’ growth in the aspects of life and existence. Some students begun to devote more attention to improving their health and had an greater awareness of the preciousness of life.

“Maybe these things taught me that, to be or not to be, that is not a question, instead, that is a truth that death has already been settled by nature.”---N16

“After so many patients’ leaving, I found that being alive had even greater value”---N15

“You have no idea what is waiting for you when waking up tomorrow, death or sunrise? Life is valued the most.”---N6

“Tell yourself to cherish life. Then, health is the most important thing, and more importantly, we should cherish the time with family and friends.”---N7

#### Professional growth

Students’ death experiences promoted the sense of responsibility for patients’ lives. The feeling of being entrusted with patients’s lives, increased their sense of professional identity as nurses.

“Responsibilities are crowding in on my mind; I think it my duty to help and save struggling lives, which is part of my job.”---N9

“I think actually, as a nurse, I could do many things for dying patients, even though just little things such as touching them or talking with them.” --- N18

### Coping

After experiencing patients’ death, nursing students talked about some methods they used to cope their feelings. There were two kinds of methods nursing students used, one was an exploratory coping method, other one was an avoidant coping method.

#### Exploratory coping method

Most students expressed their sadness, fear, and anxiety after experiencing the incident. Sharing with trusted people and writing down their experiences acted as an exploratory coping method alleviate these negative feelings.

“Of course, I’m afraid and worried. But I have to face it. Sometimes, I will talk to my friends and family about it, which helps me relax.”---N9

“...write something down to record.”---N7

#### Avoidant coping method

Contrary to exploratory coping method, avoidant coping methods means that a person does not want to be reminded stressful incidents. Some avoided contact with the dying patients and clinical situations where patients’ death had happened or was expected to occur. When the inpatients related to this situation were in the need, the nursing students would be hesitating and fearful which may affect their job performance.

“Actually, I don’t share it with others or remind it, because I don’t think it is necessary. And it’s not such a good thing.”---N18

“ I dare not go to that room but I must; so I immediately run out of the room.”---N11

“I avoided to go to the place where death happened.”--N6

#### Support

Nursing students claimed a unmet need for support and put forward some suggestions for education.

#### Clinical teachers

The students claimed that they hoped their tutors would listen to them and tell them some practical, emotional experiences and some positive coping methods, particularly when they were depressed after experiencing patients’ death. As for the authors, when we were students experienced patients’ death, we were also eager for the help of our teachers, maybe just a comfort word.

“At that time, I was in a low mood. I didn't have any desire to do anything. I just wanted the clinical teacher to listen to our interns' voices and comfort them.”---N4

“I just hope I can hear the instructors tell us how their experiences in those years helped them adjust to this process.”---N15

“I hope the teachers can say some positive words instead of saying that they are used to numbness because have more experience.”---N9

#### Education

Students called for specific training. For example, “I think schools or hospitals should arrange some courses for life and death education to teach us how to deal better with death incidents during internship and even later work.”---N16. the future.

## Discussion

The results of this study showed that the most memorable experience of a patients’ death has affected intern students in several ways, including both emotional and behavioral adjustments. First of all, emotional experience of patients’ death in our study were associated with three periods: imagining patients’ death beforehand, at the point of death, and after patients’ death. Unlike previous studies which focus on the feelings just after patients’ death, we identified the temporal aspects of that experience. While [8] (Liu and Su, 2011; Anderson et al., 2015). Three time points could help educators understand nursing students’ feelings toward death more deeply. As the topic of death in Chinese culture is surrounded by taboo, Chinese find it difficult to talk about death with each other. Nursing students feel that taboo and the fear that goes with the taboo, and feel unprepared to imagine patients’ death before entering clinical work. Actually, the emotional experience of nursing students who experienced memorable patients’ death varies with the context of the event. Hard feelings such as fear, being startled, feeling helpless, and overwhelmed by sadness were almost always expressed by student participants in our study. These feelings often occurred in situations where a patient suddenly and unexpectedly fell ill before death. These feelings are consistent with earlier researches reporting that negative feelings for death were common when facing a patient’s painful suffering [16, 26], Huang et al. 2010). These emotions occur when nursing students experienced patients’ deaths reported and while caring for dying patients [1]. The better relationship between nursing students and patients, the stronger negative emotions after patients’ death. Moreover, the age of the death patient also influences nursing students. Pity and awareness of cherished aspects of life often accompanies the death of young patients. When nursing students did something in the process of death rescue, they would deeply experience feelings of guilt after patients’ death because they thought it was their bad performance that led to patients’ death. If students just stood by, they would also feel guilty as well as helpless. In our study, the main feeling expressed frequently at all three time points was fear, an important emotion in other studies [26]. Nursing students in Western or other Asian countries with different cultures would also have negative emotions toward patients’ death. Researchers have confirmed that these negative emotions will affect nursing practice, attitudes towards death and dying [17], and even their professional identity [8, 13, 15]. The main reasons of bad emotional experiences reported by our interviewees were lack of death-related skills and knowledge.

In addition to emotions, students also had challenges regarding to cognitive, somatic, behavioral and professional aspects. Taboos about death, lack of confidence in their ability to handle death and obsessive thinking death experience made students choose to avoid contacting death and postmortem care. Students had somatizations after experiencing patients’ death which directly influenced their daily life and habits. Empathy fatigue, emotion contagion and gaps between practice and knowledge were also mentioned by nursing students. These challenges suggest that it is necessary for educators to help nursing students resolve the difficulties caused by the memorable experience of patients’ death .

Our results also found that nursing students’ death experiences were opportunities both for professional and personal growth. Unoz-Pino (2014) points out that encountering death is a significant experience for students to understand life and death. Indeed, in this study, death acted as stimuli to nursing students who would take on difficult experience, give meaning it, and then achieve growth [17, 18]. Their personal growth was evidenced by change of awareness towards health and the value of life. If students are guided appropriately, death experience could motivate their responsibility of life and further improve their career identity. Similarly, previous studies also provide evidence that encountering death of a patient can be rewarding [17].

When focusing on nursing students’ coping after experiencing death, we found two diverse coping methods. Exploratory methods can be seen as a positive way to deal with stress incidents [1]. Some students tended to share their experience with others to alleviate negative emotions. We also noticed that those students (N6/N11) who applied avoidant methods after experiencing patients’ death were more likely to have difficulties in emotion and physical symptoms. According to Edo-Gual et al. teaching nursing students how to manage their emotions is beneficial for them to deal with death in the future [13].

This study also highlighted nursing students’ great demands for supports to cope with the memorable death experience in clinical settings. However, educators in clinical settings have not realized the transformational importance of their supports for nursing students [27]. Students reported that teachers told them how to comfort patients in courses, but there was insufficient attention to the knowledge and skills need to adjust themselves to patient deaths. They expected their clinical teachers to listen to them and provide experiences relating to death and coping methods. Nursing educators played an important role in helping students pull through challenges caused by memorable patients’ death experience and promoting their growth. Similarly, previous studies evidenced that supports from clinical staff help to develop the ability of nursing students to deal with death [13, 28], and improve students’ professional growth [29, 30]. Paying more attention to death education in clinical settings could be an ideal way to improve students’ skills and attitudes [28].

Currently, Chinese nursing schools do not have specific death courses, but only cover postmortem care as a part of basic nursing or palliative courses. We found that the nursing students who had not received palliative were more likely to have negative feelings and challenges. However, most participants attending palliative courses before also had negative emotions, challenges, avoidant coping methods and need for supports which has also been reported in earlier studies [31]. This indicates that the palliative courses cannot replace the death education. Many researchers all over the world have aimed at exploring the death education method. A death education course named Problem-based learning (PBL) in Hong Kong, which provides a safe environment and lets nurses discuss and share feelings and information towards death has been proved as an effective teaching strategy [31]. Death Issues Workshop offered for registered nurses in Australia during their first year of practice greatly reduces death anxiety and increases coping skills in caring for themselves and their colleagues [32]. A death education course of 20 credit hours based on dealing with a sudden patient death conducted in China could help emergency nurses’ cope with sudden death with effective behaviors [33]. However, in China the death education is still at the theoretical level. Such education should be focused on the known vulnerabilities and needs of nurses and on proven ways of handling death in clinical practice. Nurse educators should increase the awareness of death education, pay attention to death education for nursing students in clinical practice and satisfy students’ unmet needs.

### Limitations

All our participants were female, although we tried to find male nursing students to take part in our study, so our results cannot represent male students. Through exploring nursing students’ memorable experience of death, we detected several themes including growth and coping, but could not identify the determinants of variations in those experiences. Future research should try to identify the personal and situational factors that influence the nature of death experience.

## Conclusions

Nursing students have negative emotions towards patients’ deaths. Moreover, this inevitable experience creates challenges for them in terms of irrational cognition, somatization, bad habits and professional crisis. However, these challenges can be faced with clinical teachers’ supports. Memorable death experiences are meaningful for nursing students as they encourage them to cherish life more and get a sense of professional identity. The unmet needs emerged from students’ perspectives are associated with the lack of supports from clinical tutors and shortage of death knowledge and skills. Indeed the education related to death is where the nursing profession in China needs to focus in the future. If educators can tailor effective supports and death education, these challenges can be addressed effectively.

## Supplementary Information

**Additional file 1.**

## Data Availability

The datasets generated and/or analysed during the current study are not publicly available due this study need to be continued but are available from the corresponding author on reasonable request.
