# Correction: A qualitative study of phenomenology of perspectives of student nurses: experience of death in clinical practice

**DOI:** 10.1186/s12912-022-01122-7

**Published:** 2022-12-07

**Authors:** ShiShuang Zhou, LiZhen Wei, Wei Hua, XiaoChong He, Jia Chen

**Affiliations:** 1 Department of Nursing Aministration, School of Nursing, Army Medical University, Chongqing, China; 2 XiangYa Nursing School of Central South University, 172 TongZiPou Rd, Yuelu District, Changsha, 410000 Hunan China; 3 JiangNing Hospital, Nanjing, China

**Correction: BMC Nurs 21, 74 (2022)**

**https://doi.org/10.1186/s12912-022-00846-w**

Following publication of the original article [1], the authors reported that the fourth author's name needed to be updated from “XioaChong He” to “XiaoChong He”.

The correct author name has been provided in this Correction.

The original article [1] has been corrected.
