# Supplementary material for: A qualitative study of phenomenology of perspectives of student nurses: experience of death in clinical practice
Source: BMC Nurs. 2022 Mar 29;21:74. doi: 10.1186/s12912-022-00846-w (PMC8966360; doi:10.1186/s12912-022-00846-w)
Supplement: Supplementary file 1 — Additional file 1. [file 12912_2022_846_MOESM1_ESM.docx]

| 1.Interviewer/facilitator | Which author/s conducted the interview or focus group? | page6 |
| --- | --- | --- |
| 2.Credentials | What were the researcher's credentials?E.g. PbD, MD | MD |
| 3.Occupation | What was their occupation at the time of the study? | Nursing Students |
| 4.Gender | Was the researcher male or female? | All are female |
| 5.Experience and training Relationship with participants | What experience or training did the researcher have? | Learning interview systematically |
| 6.Relationship established | Was a relationship established prior to study commencement? | No |
| 7.Participant knowledge of the interviewer | What did the participants know about the researcher? | Reasons for doing the research |
| 8.Interviewer characteristics | What characteristics were reported about the interviewer/facilitator? | Reasons and interests in the research topic |
| Domain 2:study design | |  |
| Theoretical framework |  |  |
| 9.Methodological orientation and Theory Participant selection | What methodological orientation was stated to underpin the study? | Interpretative phenomenological analysis |
| 10.Sampling | How were participants selected? | Purposive sample |
| 11.Method of approach | How were participants approached? | Face-to-face |
| 12.Sample size | How many participants were in the study? | 19 |
| 13.Non-participation Setting | How many people refused to participate or dropped out? Reasons? | None |
| 14.Setting of data collection | Where was the data collected? | Undisturbed university psychological counseling rooms |
| 15.Presence of non-participants | Was anyone else present besides the participants and researchers? | No |
| 16.Description of sample Data collection | What are the important characteristics of the sample? | Demographic data, date |
| 17.Interview guide | Interview guide Were questions, prompts, guides provided by the authors? Was it pilot tested? | Questions,Yes |
| 18.Repeat interviews | Were repeat interviews carried out? If yes, how many? | No |
| 19.Audio/visual recording | Did the research use audio or visual recording to collect the data? | Audio recording |
| 20.Field notes | Were field notes made during and/or after the interview or focus group? | Yes |
| 21.Duration | What was the duration of the interviews or focus group? | Between 60 and 90 minutes |
| 22.Data saturation | Was data saturation discussed? | Yes |
| 23.Transcripts returned | Were transcripts returned to participants for comment and/or correction? | Yes |
| Domain 3:analysis and findingsz Data analysis | |  |
| 24.Number of data coders | How many data coders coded the data? | two |
| 25.Description of the coding tree | Did authors provide a description of the coding tree? | Yes |
| 26.Derivation of themes | Were themes identified in advance or derived from the data? | Themes were derived from the data |
| 27.Software | What software, if applicable, was used to manage the data? | Nvivo |
| 28.Participant checking Reporting | Did participants provide feedback on the findings? | Yes |
| 29.Quotations presented | Were participant quotations presented to illustrate the themes/findings? Was each quotation identified? the data presented and the findings? | Yes,each quotation was identified |
| 30.Data and findings consistent | Was there consistency between the data presented and the findings? | Yes |
| 31.Clarity of major themes32.Clarity of minor themes | Were major themes clearly presented in the findings? | Yes |
| 32. Clarity of minor themes | Is there a description of diverse cases or discussion of minor themes? | Yes |
